# Supplementary material for: Optimal deep brain stimulation sites and networks for stimulation of the fornix in Alzheimer’s disease
Source: Nat Commun. 2022 Dec 14;13:7707. doi: 10.1038/s41467-022-34510-3 (PMC9751139; doi:10.1038/s41467-022-34510-3)
Supplement: Supplementary file 1 — Supplementary Information [file 41467_2022_34510_MOESM1_ESM.pdf]

Supplementary information: Optimal Stimulation Sites and Networks for Deep Brain Stimulation of the Fornix in Alzheimer's Disease

Ana Sofía Ríos[1], Simón Oxenford[1], Clemens Neudorfer[1], Konstantin Butenko[1], Ningfei Li[1], Nanditha Rajamani[1], Alexandre Boutet[2,3,4], Gavin J.B. Elias[2,3], Jurgen Germann[2,3], Aaron Loh[2,3], Wissam Deeb[5,6], Fuyixue Wang[7,8], Kawin Setsompop[7,8,9], Bryan Salvato[10], Leonardo Almeida[11], Kelly D. Foote[11], Robert Amaral[12], Paul B. Rosenberg[13], David F. Tang-Wai[14,3], David A. Wolk[15], Anna D. Burke[16], Stephen Salloway[17,18], Marwan N. Sabbagh[16], M. Mallar Chakravarty[12,19,20], Gwenn S. Smith[13], Constantine G. Lyketsos[13], Michael S. Okun[11], William S. Anderson[21], Zoltan Mari[21,22], Francisco A. Ponce[16], Andres M. Lozano[2,3], Andreas Horn*[1,23,24]

Affiliations

1. Movement Disorder and Neuromodulation Unit, Department of Neurology, Charité – Universitätsmedizin Berlin, corporate member of Freie Universität Berlin and Humboldt-Universität zu Berlin, Berlin, Germany.
2. Division of Neurosurgery, Department of Surgery, University Health Network and University of Toronto, Toronto, M5T2S8, Canada
3. Krembil Research Institute, University of Toronto, Toronto, M5T2S8, Canada
4. Joint Department of Medical Imaging, University of Toronto, Toronto, M5T1W7, Canada
5. UMass Chan Medical School, Department of Neurology, Worcester, MA 01655
6. UMass Memorial Health, Department of Neurology, Worcester, MA 01655
7. Athinoula A. Martinos Center for Biomedical Imaging, Department of Radiology, Harvard Medical School, Massachusetts General Hospital, Charlestown, MA, USA
8. Harvard-MIT Health Sciences and Technology, MIT, Cambridge, MA, USA
9. Department of Radiology, Stanford University, CA, USA
10. University of Florida Health Jacksonville, Jacksonville, FL, USA
11. Norman Fixel Institute for Neurological Diseases, Departments of Neurology and Neurosurgery, University of Florida, Gainesville, FL
12. Cerebral Imaging Centre, Douglas Research Centre, Montreal QC, Canada
13. Department of Psychiatry and Behavioral Sciences and Richman Family Precision Medicine Center of Excellence, School of Medicine, Johns Hopkins University, Baltimore, MD, USA
14. Department of Medicine, Division of Neurology, University Health Network and University of Toronto, Toronto, M5T2S8, Canada
15. Department of Neurology, University of Pennsylvania, Philadelphia, PA, USA
16. Barrow Neurological Institute, Phoenix, AZ
17. Department of Psychiatry and Human Behavior and Neurology, Alpert Medical School of Brown University, Providence, RI, USA
18. Memory & Aging Program, Butler Hospital, Providence, USA

19. Department of Psychiatry, McGill University, Montreal, QC, Canada
20. Biological and Biomedical Engineering, McGill University, Montreal, QC, Canada
21. Johns Hopkins School of Medicine, Baltimore, MD, USA
22. Cleveland Clinic Lou Ruvo Center for Brain Health, Las Vegas, NV, USA
23. Center for Brain Circuit Therapeutics, Department of Neurology, Brigham and Women's Hospital, MA, USA
24. Departments of Neurology and Neurosurgery, Massachusetts General Hospital, MA, USA

*** Corresponding Author**

Andreas Horn, MD, PhD

Associate Professor of Neurology, Center for Brain Circuit Therapeutics, Department of Neurology, Brigham and Women's Hospital, MA, USA. E-mail: ahorn1@bhw.harvard.edu

Keywords

Alzheimer's disease (AD), Deep Brain Stimulation (DBS), Fornix, Connectome, Structural Connectivity, Network mapping

Supplementary Table 1. Inclusion/exclusion criteria of Toronto-based pilot clinical trial (NCT00658125).

Inclusion Criteria	Exclusion Criteria
1. Man or woman aged 40 to 80 years old	1. Pre-existing structural brain abnormalities (such as tumor, infarction, or intracranial hematoma)
2. Satisfies the diagnostic criteria for probable AD*	2. Other neurologic or psychiatric diagnoses
3. Has received the diagnosis of AD within the past 2 years	3. Medical comorbidities that would preclude patients from undergoing surgery
4. Has a Clinical Dementia Rating (CDR) score of 0.5 or 1.0	
5. Has a score between 18 and 28 on the Mini Mental State Examination (MMSE)	
6. Has been taking a stable dose of cholinesterase inhibitors for a minimum of 6 months	

*See McKhann G, Drachman D, Folstein M, et al. Clinical diagnosis of Alzheimer's disease: report of the NINCDS-ADRDA Work Group under the auspices of Department of Health and Human Services Task Force on Alzheimer's Disease. Neurology 1983;34: 939–944.

Supplementary Table 2. Inclusion/exclusion criteria of ADvance multi-centre trial (NCT0160806).

Inclusion Criteria	Exclusion Criteria
1. 45 years of age (inclusive).	1. Must meet certain criteria on cognitive and behavioral scales.
2. Probable Alzheimer's disease according to the National Institute of Aging Alzheimer's disease Association criteria.	2. Current major psychiatric disorder such as schizophrenia, bipolar disorder or major depressive disorder based on psychiatric consult at screening visit.
3. Must meet certain criteria on cognitive and behavioral rating scales.	3. History of head trauma in the 2 years prior to signing the consent to participate in the study.
4. If female, subjects who are post-menopausal or surgically sterile or willing to use birth control methods for the duration of the study.	4. History of brain tumor, subdural hematoma, or other clinically significant (in the judgement of the investigator) space-occupying lesion on CT or MRI.
5. An available caregiver willing to participate.	5. Active psychiatric disorder.
6. Subject is living at home and likely to remain at home for the study duration.	6. Mental retardation.

Inclusion Criteria	Exclusion Criteria
7. The subject is currently taking a stable dose of cholinesterase inhibitor (AChEI) medication for at least 60 days.	7. Current alcohol or substance abuse as defined by Diagnostic and Statistical Manual of Mental Disorders, Fourth Edition, Text Revision (DSM-IV-TR).
	8. Contraindications for PET scanning (e.g., insulin dependent diabetes).
	9. Contraindications for MRI scanning, including implanted metallic devices (e.g., non-MRI-safe cardiac pacemaker or neurostimulator; some artificial joints metal pins; surgical clips; or other implanted metal parts), or claustrophobia or discomfort in confined spaces.
	10. Abnormal lab results that, in the opinion of the investigator and/or enrollment review committee, would preclude participation in the study.
	11. Abnormal cardiovascular or neurovascular disorder that, in the opinion of the investigator and/or enrollment review committee, would preclude participation in the study.
	12. Unstable doses of any medication prescribed for the treatment of memory loss or Alzheimer's disease.
	13. Currently prescribed any non-AD medications that, in the opinion of the investigator and/or enrollment committee, would preclude participation in the study.
	14. Is unable or unwilling to comply with protocol follow-up requirements.
	15. Has a life expectancy of < 1 year.
	16. Is actively enrolled in another concurrent clinical trial.

Supplementary Table 3. Demographic and clinical data of the patients included. Clinical outcomes measured by Alzheimer's Disease Assessment Scale 11 – cognitive subscale (ADAS-cog 11). Absolute change calculated subtracting 12-month ADAS-cog 11 value from Baseline ADAS-cog 11. Relative change calculated by dividing absolute changes by pre-operatory score and multiplying by 100.

**Patient ID**	**Age at Diagnosis (years)**	**Sex**	**Baseline ADAS-cog 11**	**12-month ADAS-cog 11**	**Absolute change** (pre-post)	**Relative change** (Absolute change)/pre *100	**Group**
01	62	Female	28	34	-6	-21.42	Poor responders
02	77	Female	22	30	-8	-36.36	Poor responders
03	76	Male	19	34	-5	-78.94	Poor responders
04	65	Female	17	39	-22	-129.41	Poor responders
05	50	Female	19	21	-2	-10.52	Middle responders
06	66	Male	13	18	-5	-38.46	Poor responders
07	64	Male	13	15	-2	-15.38	Middle responders
08	60	Male	24	31	-7	-29.16	Poor responders
09	72	Male	23	30	-7	-30.43	Poor responders
10	72	Male	13	24	-11	-84.61	Poor responders
11	62	Male	12	7	5	41.67	Top responders
12	69	Female	15	24	-9	-60	Poor responders
13	67	Female	31	36	-5	-16.13	Middle responders
14	60	Male	29	43	-14	-48.28	Poor responders
15	67	Male	19	26	-7	-36.84	Poor responders
16	52	Female	32	33	-1	-3.13	Middle responders
17	75	Male	16	29	-13	-81.25	Poor responders
18	68	Female	18	23	-5	-27.78	Poor responders
19	72	Female	23	24	-1	-4.35	Middle responders
20	58	Female	15	36	-11	-140	Poor responders
21	47	Male	22	10	12	54.55	Top responders
22	61	Male	16	19	-3	-18.75	Middle responders
23	73	Female	16	22	-6	-37.5	Poor responders
24	69	Female	21	42	-21	-100	Poor responders
25	74	Female	17	30	-13	-76.48	Poor responders

Patient ID	Age at Diagnosis (years)	Sex	Baseline ADAS-cog 11	12-month ADAS-cog 11	Absolute change (pre-post)	Relative change (Absolute change)/pre *100	Group
26	72	Female	24	29	-5	-20.83	Middle responders
27	69	Male	28	38	-10	-35.71	Poor responders
28	66	Male	14	15	-1	-7.14	Middle responders
29	68	Male	20	28	-8	-40	Poor responders
30	74	Female	16	15	1	6.25	Top responders
31	66	Female	17	12	5	29.41	Top responders
32	57	Female	35	51	-16	-45.71	Poor responders
33	57	Female	22	39	-17	-72.27	Poor responders
34	72	Male	21	23	-2	-9.52	Middle responders
35	58	Female	17	35	-18	-105.88	Poor responders
36	73	Female	22	18	4	18.18	Top responders
37	72	Female	19	19	0	0	Middle responders
38	77	Male	18	17	1	5.56	Top responders
39	71	Female	16	15	1	6.25	Top responders
40	79	Male	13	21	-8	-61.54	Poor responders
41	74	Male	18	17	1	5.56	Top responders
42	76	Male	11	17	-6	-54.55	Poor responders
43	59	Female	21	22	-1	-4.76	Middle responders
44	51	Male	13	40	-27	-207.69	Poor responders
45	77	Male	10	19	-9	-90	Poor responders
46	71	Male	22	19	3	13.64	Top responders

Normative Connectomes: Underlying Data

Supplementary Table 4. Specification of normative connectome data. Abbreviations: TR = Repetition time, TE = Echo time, FOV = Field of view, BOLD = Blood oxygenation level-dependent, EPI = Gradient-echo echo-planar imaging, FA = Flip angle

Connectome	Scan parameters	References	Data sources
Structural: *In vivo* human whole-brain Connectom diffusion MRI dataset at 760 μm isotropic resolution	Scanner: MGH-USC 3T Connectom.<br>Maximum gradient strength of 300mT/m and maximum slew rate of 200 T/m/s, custom-built 64-channel phased-array coil.<br>gSlider-SMS sequence.<br>gSlider encoding: 5<br>MB factor: 2<br>$R_{inplane}$ factor: 3<br>Acquisition: Axial (PE along AP/PA)<br>TR/TE: 3500/75 ms<br>FOV: 220.0 ∗ 218.5 mm<br>Acquisition matrix: 290 ∗ 288<br>Acquired slices: 190<br>Slice thickness: 0.76 mm<br>Effective echo spacing: 0.34 ms<br>Readout bandwidth: 1150 Hz/Pixel<br>Phase partial Fourier: 6/8<br>b-values: 1000, 2500 s/mm^2<br>144 (b0), 420 (b1000), 840 (b2500) w/AP/PA (total 2808 volumes)<br>Total acquisition time: ~14.5 hours	Wang et al. (2021) Sci. Data[1]	9 two-hour scan sessions<br>1 healthy subject
**Functional**: The organization of the human cerebral cortex estimated by intrinsic functional connectivity	Scanner: 3T Tim Trio scanners (Siemens, Erlangen, Germany) 12-channel receive coil array, Gradient -echo echo-planar imaging (EPI) sequence sensitive to BOLD contrast.<br>Acquisition: Slices aligned to anterior commissure-posterior commissure plane<br>EPI parameters<br>TR/TE: 3000 ms/30 ms<br>FA: 85°, 3 * 3 * 3-mm voxels<br>FOV: 216<br>47 axial slices collected with intervaled acquisition, no gap between slices<br>6.2 minute-functional run (124 timepoints)	Yeo et al. (2011) J. Neurophysiol[2]<br><br>Holmes et al. (2015) Sci. Data[3]	Resting-state fMRI data from 1,000 health subjects (average 1.7 runs per subject)

Final model parameters of DBS fiber filtering

During the training phase of model optimization in the DBS fiber filtering analysis, a variety of parameters were tested with the aim to create a tract-set that was i) robustly predictive during cross-validation within the training set (leave-one-out and several k-fold strategies were interactively tested) and ii) was *not robust* to permutations of improvement data (also see fig. 4). The following set of parameters were finally selected and used to cross-predict outcomes in the test-cohort.

Supplementary Table 5. Model parameters available in the DBS fiber filtering tool implemented in Lead-DBS, their units, range, and selected value, as well as a brief explanation of what the parameter means.

Model parameter	Unit	Range	Selected Value	Explanation
Model Setup	N/A	[Sum, Mean, Peak, 5% Peak]	Peak	The peak value of each E-field and each Tract was considered
Correlation Type	N/A	[Pearson, Spearman, Bend]	Spearman	Spearman's rank correlations were used (see methods: Model considerations)
Tracts "connected" if peak E-field magnitude they traverse is above	V/m	0.05 – 2.5	0.36 V/m	(see next parameter)
Tracts must be connected to > x % of E-fields	%	0 – 100	20%	Tracts were only considered if they traversed regions with > 0.36 V/m in > 20 % E-fields
Show/Use number of Fibers	%	0 – 100	70%	70% of fibers with positive R-values were visualized and used in predictive models (cross-validations within training cohort and training > test cross-predictions)
Base Prediction On	N/A	[Sum, Mean, Peak, 5% Peak, Profile of Scores: Pearson, Profile of Scores: Spearman, Profile of Scores: Bend]	Profile of Scores: Spearman	This setting was used since it follows the same logic as in sweetspot and network mapping approaches also used here.

Sweetspot Coordinates

Supplementary Table 6. Probabilistic Stimulation Mapping peak and center coordinates in non-mirrored and mirrored data.

	**Sweetspot**		**Sourspot**	
	Unflipped analysis			
	**LH: 552 voxels, 14.9 mm³**	**RH: 267 voxels, 7.09 mm³**	**LH: 516 voxels, 13.93 mm³**	**RH: 892 voxels, 24.08 mm³**
**Peak coordinate**	X = -3.9 mm<br>Y = -1.5 mm<br>Z = -3.6 mm	X = 3 mm<br>Y = -0.9 mm<br>Z = -3 mm	X = -5.4 mm<br>Y = -0.3 mm<br>Z = -6.9 mm	X = 4.5 mm<br>Y = 2.1 mm<br>Z = -4.2 mm
**Cluster center**	X = -5.1 mm<br>Y = 0.9 mm<br>Z = -3.3 mm	X = 2.4 mm<br>Y = -0.3 mm<br>Z = -3 mm	X = -4.2 mm<br>Y = 0 mm<br>Z = -6.9 mm	X = 3.9 mm<br>Y = 0.9 mm<br>Z = -5.7 mm
	Flipped Analysis			
	**LH: 392 voxels, 10.58 mm³**	**RH: 586 voxels, 15.82 mm³**	**LH: 734 voxels, 19.81 mm³**	**RH: 797 voxels, 21.51 mm³**
**Peak coordinate (fig. 4)**	X = -3.9 mm<br>Y = -1.5 mm<br>Z = -3 mm	X = 6.9 mm<br>Y = 0 mm<br>Z = -5.1 mm	X = -5.1 mm<br>Y = 2.4 mm<br>Z = -4.8 mm	X = 1.5 mm<br>Y = 0.3 mm<br>Z = -6.9 mm
**Cluster center (fig. 4)**	X = -4.8 mm<br>Y = -0.9 mm<br>Z = -3.6 mm	X = 3.9 mm<br>Y = 1.2 mm<br>Z = -3.3 mm	X = -4.5 mm<br>Y = 0 mm<br>Z = -6.6 mm	X = 3.6 mm<br>Y = 0.3 mm<br>Z = -6.3 mm

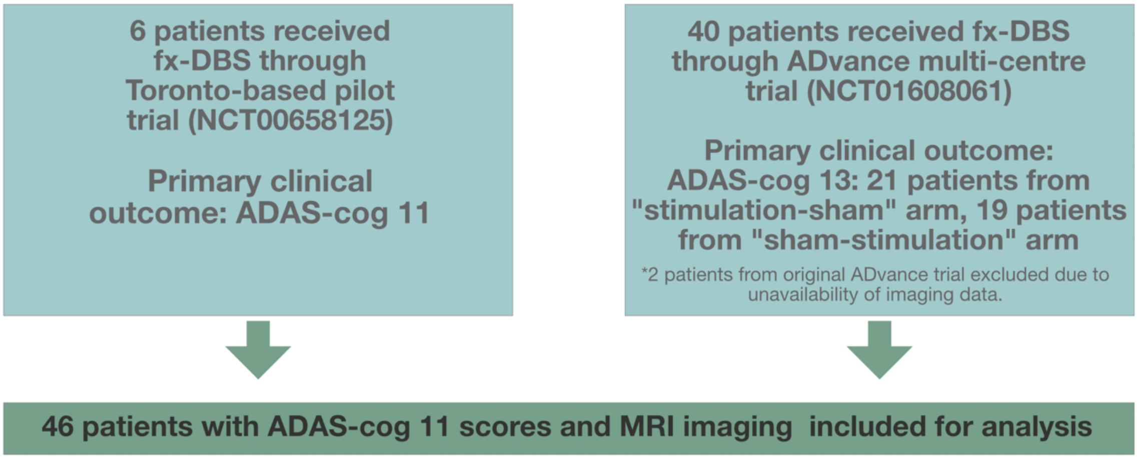

Supplementary Figure 1. Flowchart summarizing patient inclusion for this work.

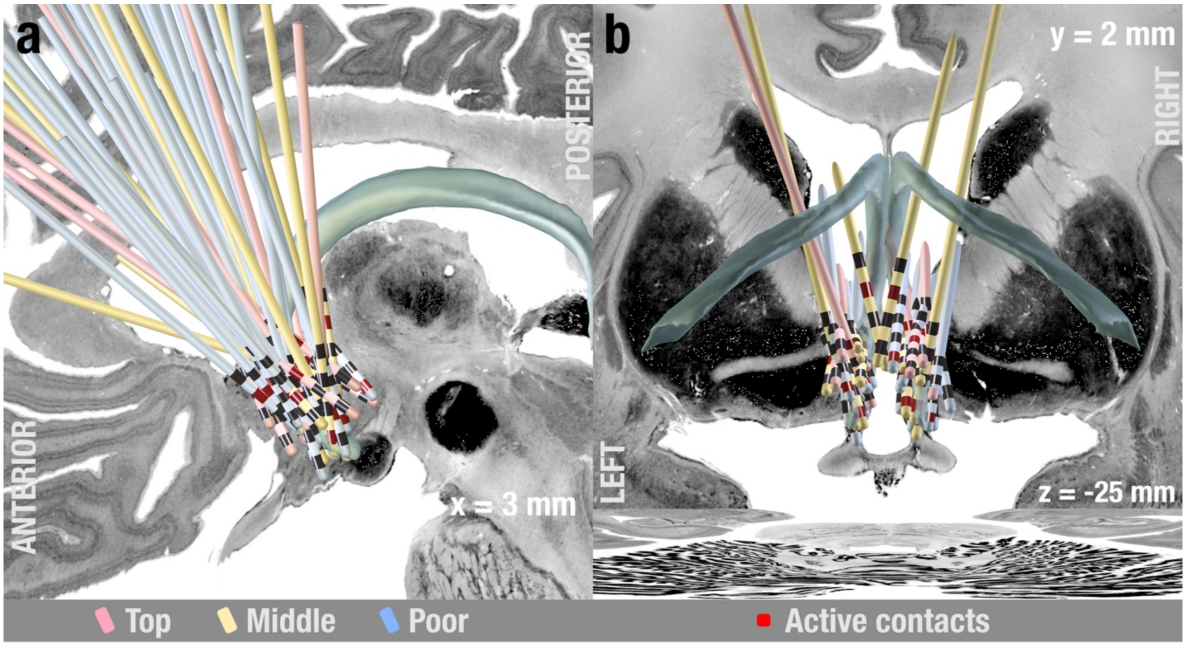

Supplementary Figure 2. Electrode localizations. a) Sagittal and b) coronal view showing solid electrodes of the 46 patients included in this study, classified by outcome group (blue-poor responders, yellow-middle responders, pink-top-responders), active contacts highlighted with red superimposed on slices of a brain cytoarchitecture atlas in MNI 152 space[4]. Fornix informed by the CoBrALab Atlas [5].

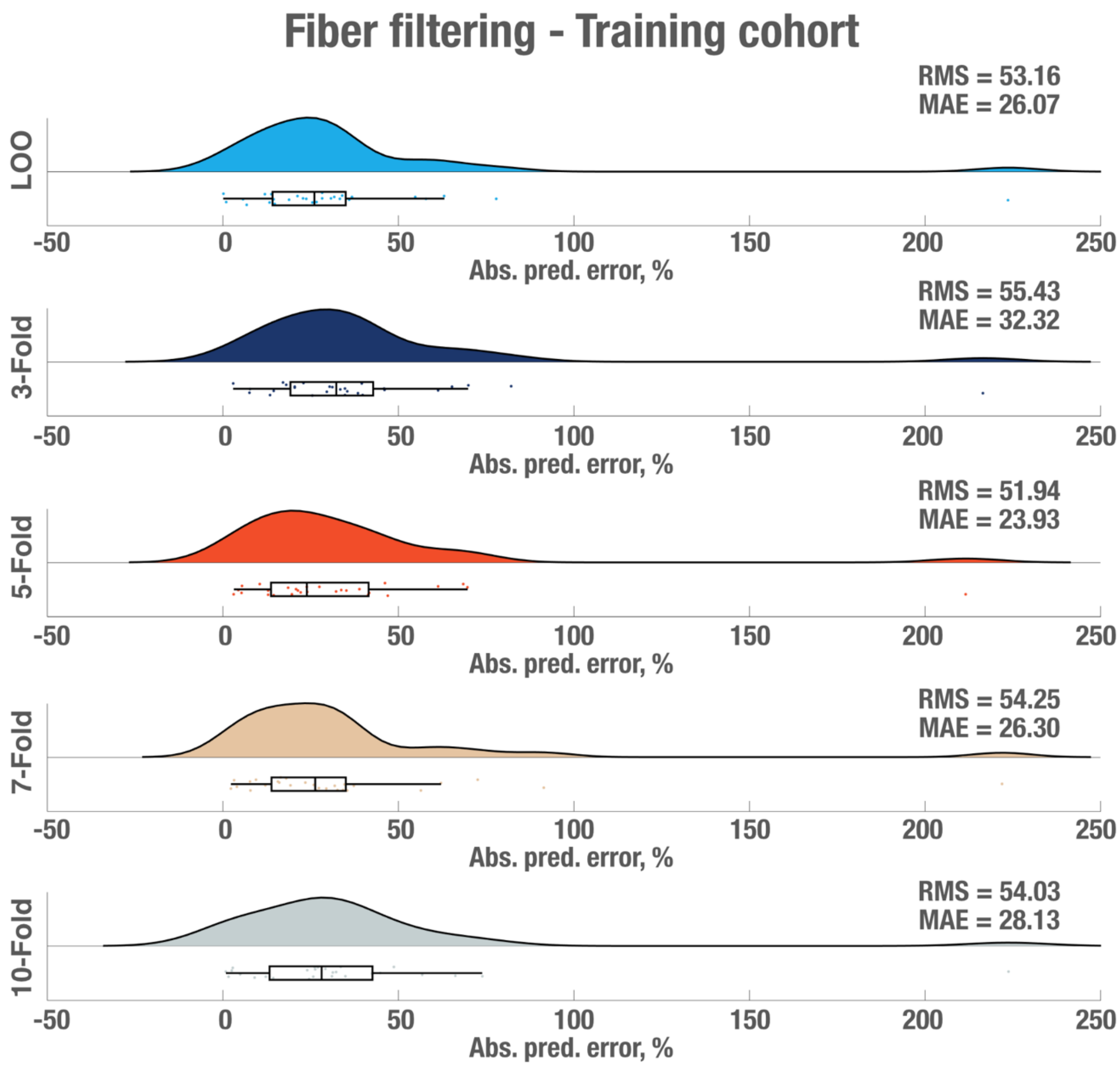

Supplementary Figure 3. In-fold analysis from fiber filtering analysis on Training cohort (N = 28) showing absolute predicted error, root mean square deviation (RMS) and median absolute error (MAE) for each of the validation approaches. The boxplot displays the interquartile range in the box with the median percentual absolute predicted error as a vertical line, whiskers extend to 1.5 times the interquartile range, outlier points outside of this range are plotted. LOO: Leave one out.

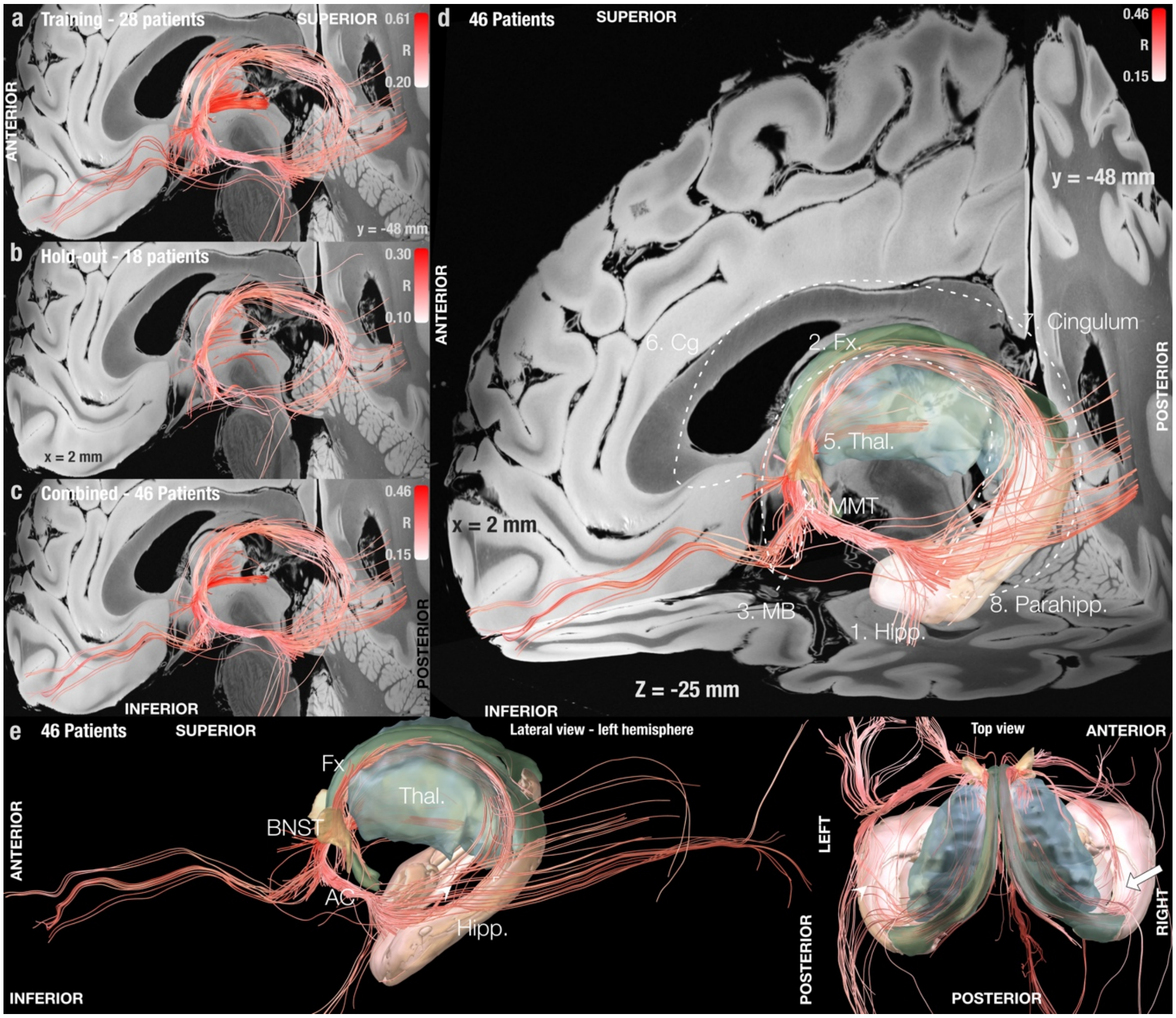

Supplementary Figure 4. Fiber tracts associated with optimal clinical response superimposed on slices of a 100-μm, 7T brain scan in MNI 152 space. From a set of 5 million fiber tracts sampled from a high-resolution connectome, the ones preferably modulated by top-responding (and not by poor-responding) patients were selected using the DBS fiber filtering method and visualized. The process was repeated on the training-cohort (N = 28) (a), the test-cohort (N = 18) (b), and both cohorts combined (N = 46) (c). Fiber tracts are color-coded by the resulting Spearman's rank correlation coefficients (R-values) which shows how strongly modulating each bundle correlated with clinical response across patients. d) Results from panel c superimposed on atlas structures forming part of the circuit of Papez, also visualized by dotted arrows. e) Lateral and top views of fibertract superimposed with structures of interest, white arrow indicates intersection of streamlines of the fornix and the anterior commissure that could give the illusion of a loop on lateral projection views. 1. Hipp: Hippocampus, 2. Fx: Fornix, 3. MB: mamillary bodies, 4. MMT: mamillothalamic tract, 5. Thal: thalamus, 6. Cg: Cingulate gyrus, 7. Cingulum and 8. Parahipp: Parahippocampal gyrus, BNST: bed nucleus of the stria terminalis. The backdrop features an ultra-high resolution (100 μm) template of the human brain[6]. Structures: Fornix (blue-green), Hippocampus (pink), Thalamus(blue) informed by the CoBrALab Atlas[5], Bed nucleus of the stria terminalis (light brown) informed by the Atlas of the Human Hypothalamus[7].

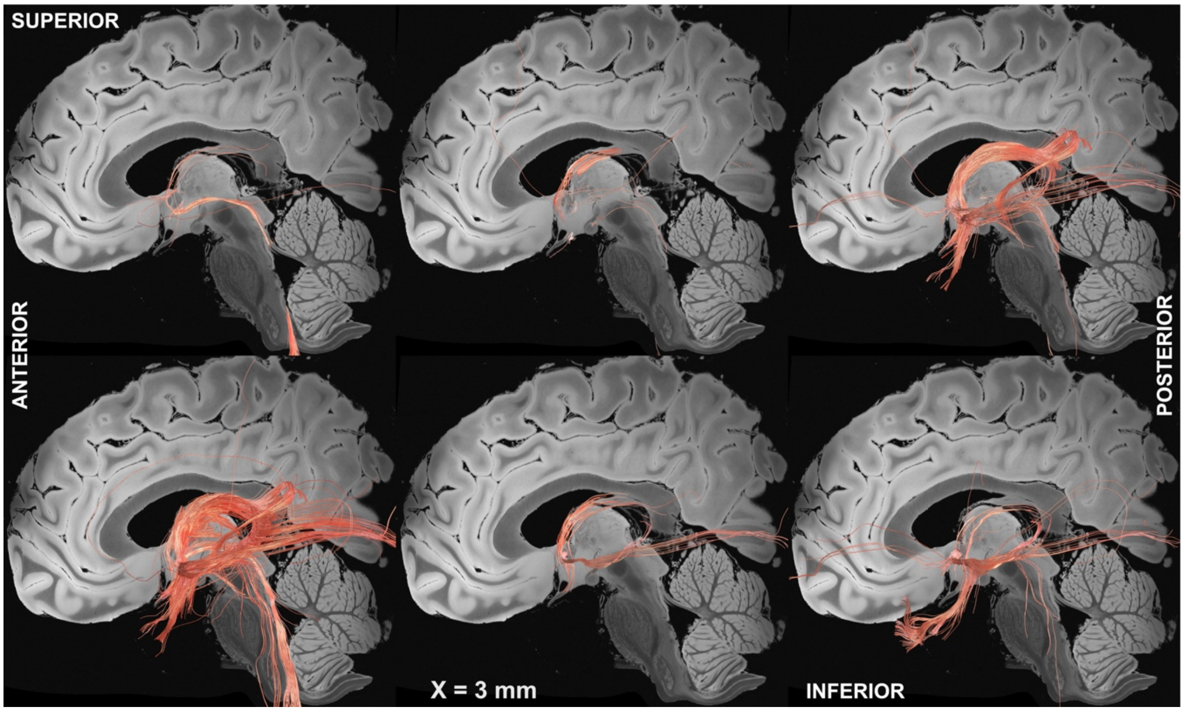

Supplementary Figure 5. Random permutation results. Example results when repeating the DBS fiber filtering method after randomly permuting clinical improvement values across patients (N = 46). This process was performed to demonstrate that tract results do not merely reflect average connectivity of the group of DBS electrodes but are highly informed by improvement values. For instance, the result on the top left highlights a connection to the brainstem, the one in the top middle the stria terminalis and the one on the bottom middle and right panels the anterior commissure. The backdrop features an ultra-high resolution (100 μm) template of the human brain[6]

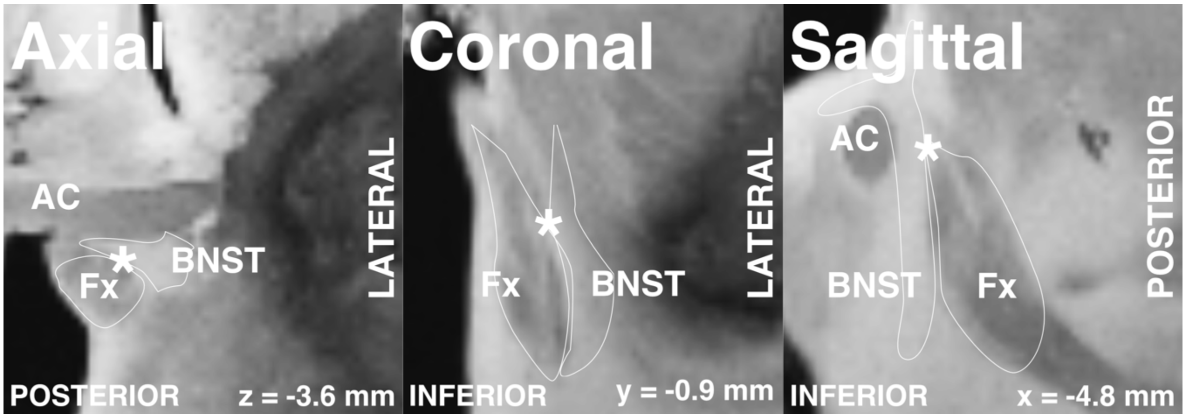

Supplementary Figure 6. Close up view of positive sweetspot cluster center coordinate at the junction between fornix and bed nucleus of stria terminalis (BNST). Fx: Fornix, AC: Anterior Commissure. The backdrop features an ultra-high resolution (100 μm) template of the human brain[6]

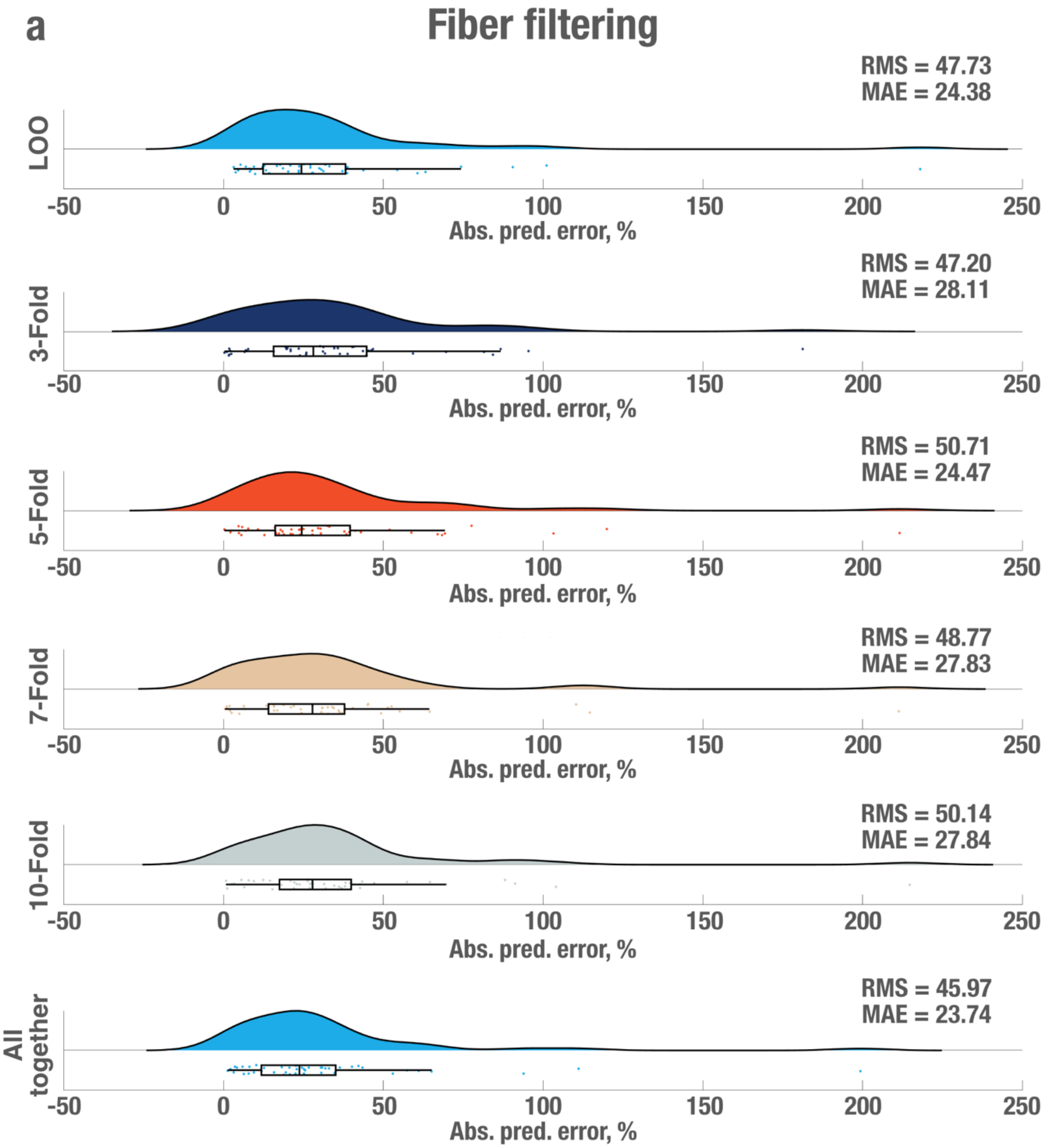
a
Fiber filtering
RMS = 47.73
MAE = 24.38
LOO
RMS = 47.20
MAE = 28.11
3-Fold
RMS = 50.71
MAE = 24.47
5-Fold
RMS = 48.77
MAE = 27.83
7-Fold
RMS = 50.14
MAE = 27.84
10-Fold
RMS = 45.97
MAE = 23.74
All together
-50 0 50 100 150 200 250
Abs. pred. error, %

Continuation Figure S7

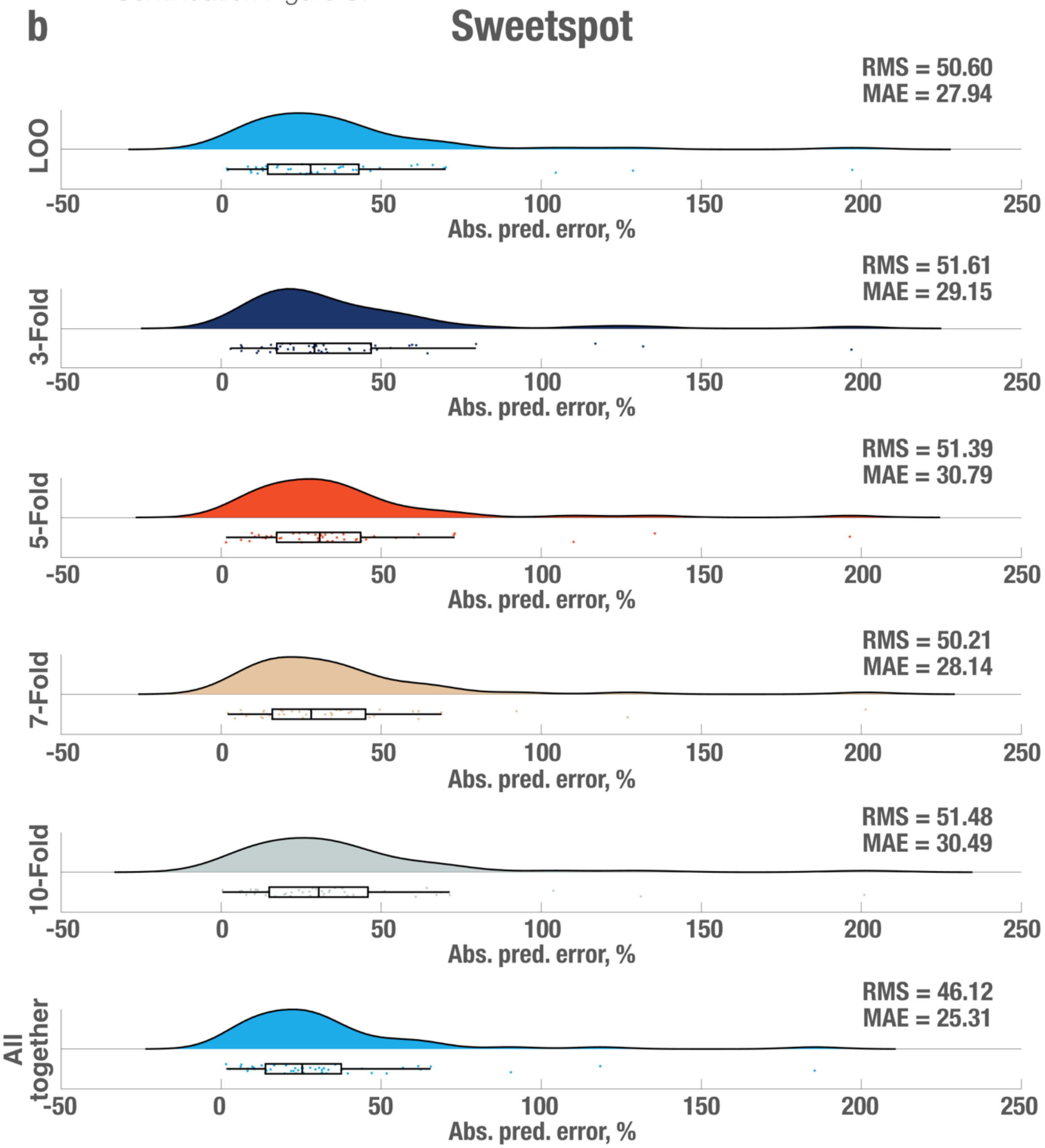

Continuation Figure S7

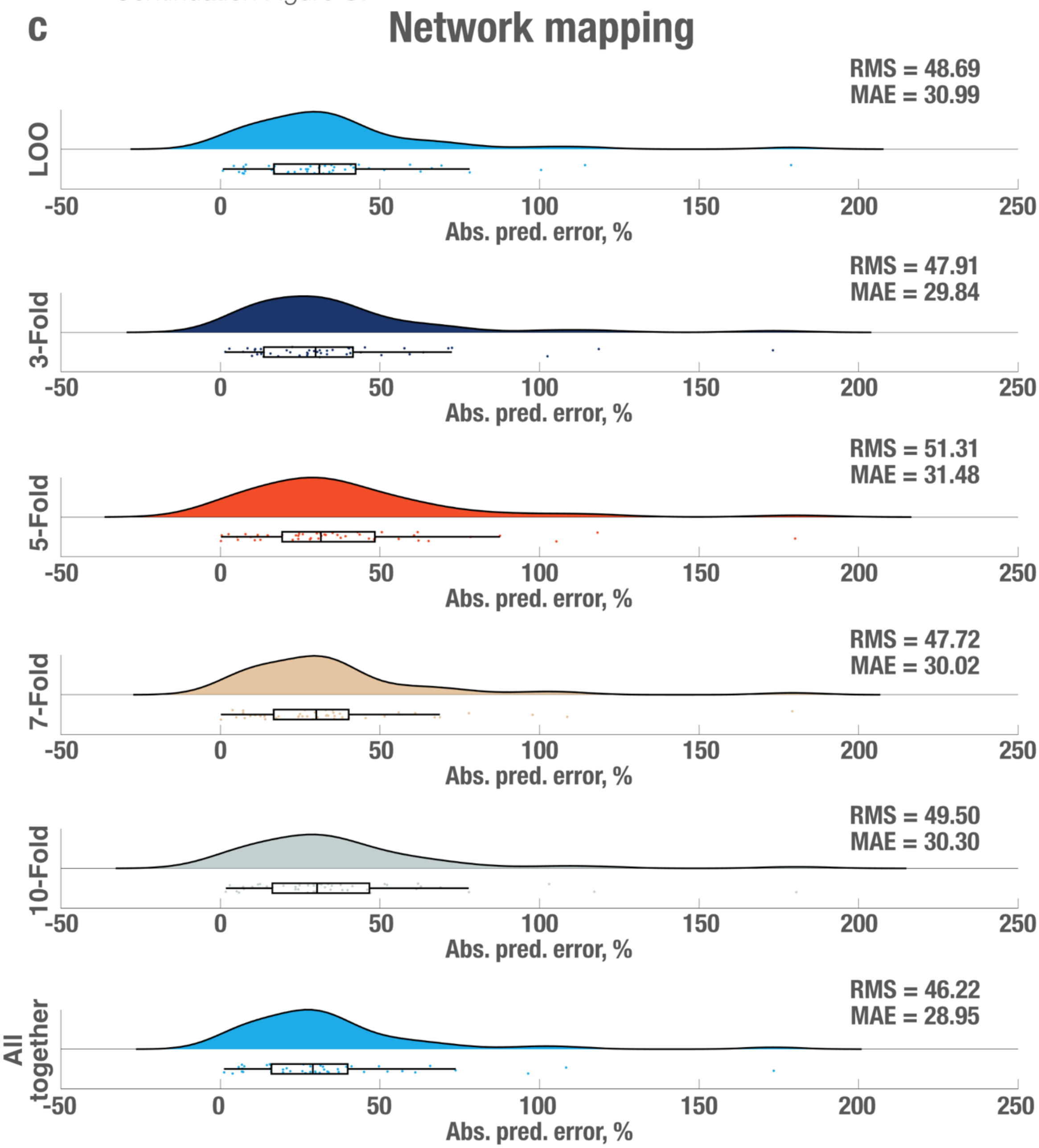

Supplementary Figure 7. In-fold analysis from summary showing absolute predicted error, root mean square deviation (RMS) and median absolute error (MAE) for each of the validation approaches followed on fiber filtering (a), sweetspot (b) and network mapping (c) methods in the entire cohort (N = 46). The boxplot displays the interquartile range in the box with the median percentual absolute predicted error as a vertical line, whiskers extend to 1.5 times the interquartile range, outlier points outside of this range are plotted. LOO: Leave one out.

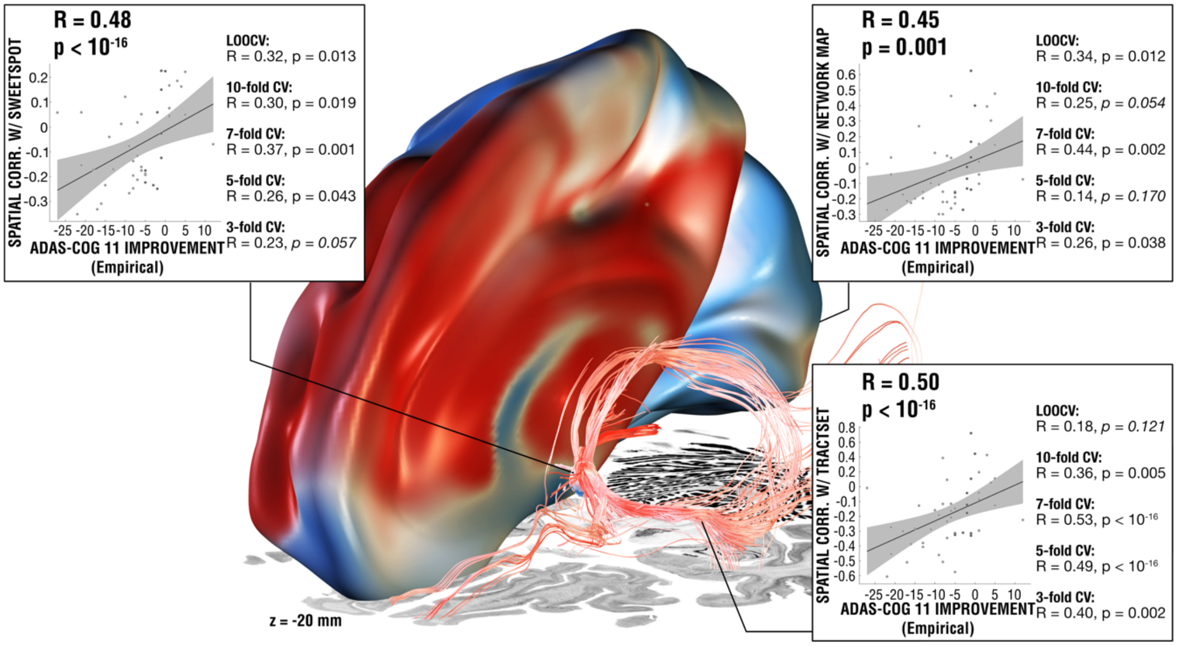

Supplementary Figure 8. Results summary including sweetspot, tract- and network-level models calculated with absolute ADAS-cog 11 outcomes. Three levels of analysis led to mostly significant predictions of clinical outcomes across leave-one-patient-out (LOO) and multiple k-fold designs, plots show the fitting of a linear model representing the degree to which, stimulating voxels (left), functional regions (top-right) and tracts (bottom-right) explains variance in clinical outcomes across the 46 patients using Spearman correlation, gray shaded areas represent 95% confidence intervals. Three level analysis results were superimposed on slices of a brain cytoarchitecture atlas in MNI 152 space[4]. RMS: Root mean square deviation, MAE: Median absolute error.

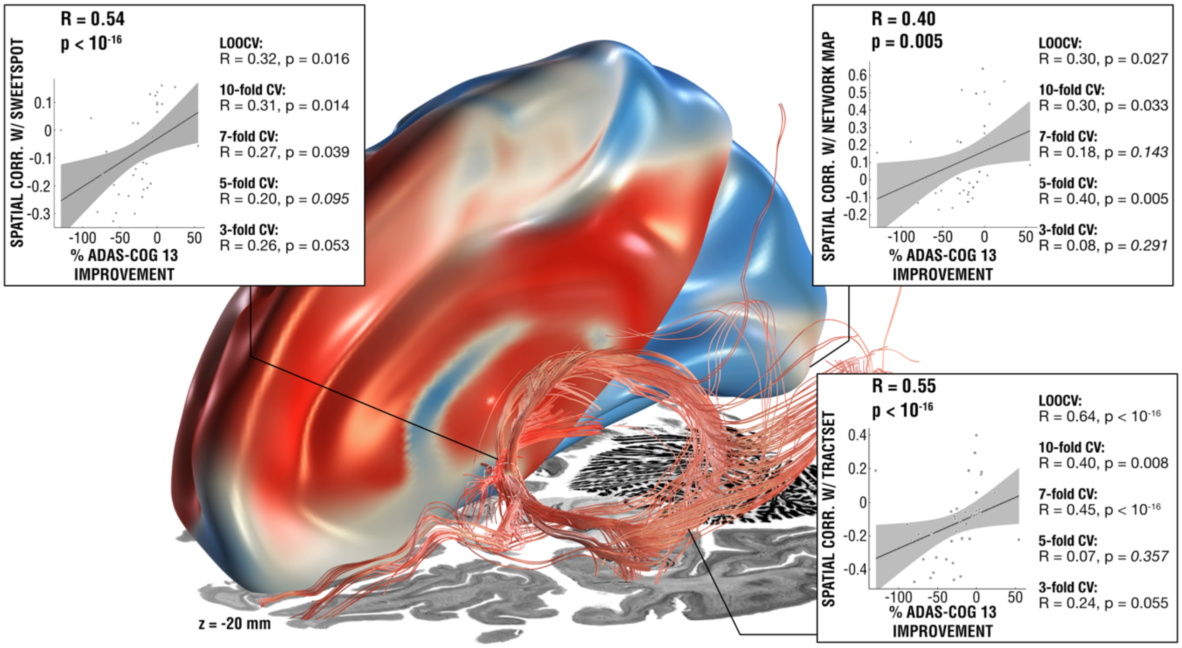

Supplementary Figure 9. Results summary including sweetspot, tract- and network-level models calculated with ADAS-cog 13 outcomes (available for ADvance trial patients, N = 40). Three levels of analysis led to mostly significant predictions of clinical outcomes across leave-one-patient-out (LOO) and multiple k-fold designs, plots show the fitting of a linear model representing the degree to which stimulating voxels (left), functional regions (top-right) and tracts (bottom-right) explains variance in clinical outcomes across the 40 patients using Spearman correlation, gray shaded areas represent 95% confidence intervals. Three level analysis results were superimposed on slices of a brain cytoarchitecture atlas in MNI 152 space[4]. RMS: Root mean square deviation, MAE: Median absolute error.

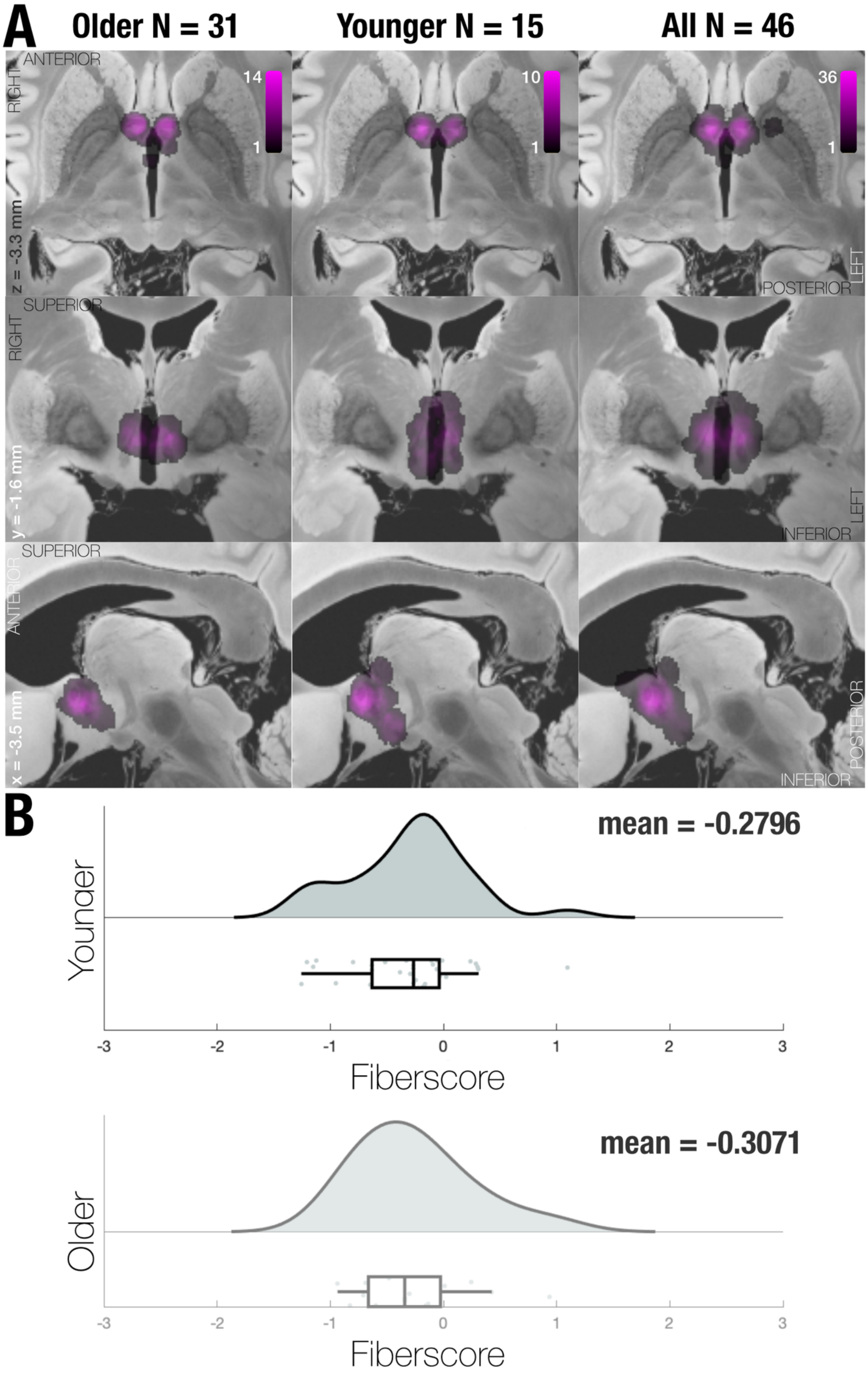

A
Older N = 31
Younger N = 15
All N = 46
ANTERIOR
RIGHT
14
1
10
1
36
1
z = -3.3 mm
POSTERIOR
LEFT
SUPERIOR
RIGHT
y = -1.6 mm
INFERIOR
LEFT
SUPERIOR
ANTERIOR
x = -3.5 mm
POSTERIOR
INFERIOR
B
mean = -0.2796
Younger
-3
-2
-1
0
1
2
3
Fiberscore
mean = -0.3071
Older
-3
-2
-1
0
1
2
3
Fiberscore

Supplementary Figure 10. Effects of Age. a) Axial, coronal, and sagittal overlay of maps created from stimulation volumes of subjects older than 65 years (left), younger than 65 years (middle) and whole cohort (right), color bar representing amount of overlapping stimulation volumes. b) Fiberscores obtained through DBS fiber filtering analysis explained in Methods and Results sections, by the stimulation volumes of younger than 65-year-old patients (top, N = 15), and patients 65-year-old or older (bottom, N = 31), p(T-test) = 0.790. The model used to estimate these scores was calculated in a leave-one-patient out design across the entire cohort. The boxplot displays the interquartile range in the box with the mean fiberscore as a vertical line, whiskers extend to 1.5 times the interquartile range, outlier points outside of this range are plotted. The backdrop features an ultra-high resolution (100 μm) template of the human brain[6]

Supplementary Methods

Narrative section of methods / predictive models:

In all three models, each patient contributed their relative improvement of ADAS-cog-11 scores (before surgery, one year after surgery).

Beyond that, each model (i) tracts, ii) sweetspots and iii) functional networks) was run independently from one another.

- i) For tracts, each patient contributed the peak E-field amplitude that each tract of the normative connectome was modulated by.
- ii) For sweetspots, each patient contributed the modeled electric field in MNI space (represented as a NIfTI volume).
- iii) For functional networks, each patient contributed a (normative) rs-fMRI map seeding from the individual patient ("connectivity fingerprints").

Then, the three models created a i) combination of tracts ii) optimal target (sweetspot), and iii) functional network profile associated with optimal clinical improvements.

- i) For tracts, this was achieved by rank correlating the modulation amplitude imposed on each tract with clinical improvements across the set of patients. This led to an R-value for each tract, denoting how well its modulation correlated with clinical improvements (the concept was introduced in Irmen et al. 2019 Annals of Neurology).
- ii) For sweetspots, this was achieved by rank correlating each voxel with clinical outcomes across the set of patients. This led to an R-map denoting how well modulations of specific voxels correlated with clinical outcomes (the concept was introduced in Horn et al. 2022 PNAS).
- iii) For functional networks, this was achieved by correlating the voxel values of connectivity fingerprints with clinical improvements across the set of patients. This led to an R-map denoting how well connectivity estimates between stimulation sites and each voxel in the brain correlated with clinical outcomes (the concept was introduced in Horn et al. 2017 Annals of Neurology).

Finally, data was cross-validated within the three models:

- i) For tracts, this was achieved by rank correlating the impacts of the E-Fields of an unseen patient on all tracts and their R-values This led to a fiberscore denoting how specifically an unseen E-Field modulated tracts associated with optimal outcomes (the concept was introduced in Horn et al. 2022 PNAS).
- ii) For sweetspots, this was achieved by spatially correlating the E-Fields of an unseen patient with the R-map model. This led to a sweetspot score denoting correlation coefficients of agreement between the actual stimulation field and an "optimal" stimulation field (represented by the R-map; the concept was introduced in Horn et al. 2022 PNAS).
- iii) For functional networks, this was achieved by spatially correlating the functional connectivity fingerprints with the R-map model. This led to a network score denoting correlation coefficients of agreement between the actual network profile and an optimal network profile (represented by the R-map; the concept was introduced in Horn et al. 2017 Annals of Neurology).

Data Availability

Anonymized derivatives of stimulation data used for the described analyses are openly available on OSF (https://osf.io/bckuf). The resulting tract atlas, sweet spot and fMRI network pattern are openly available within Lead-DBS software (www.lead-dbs.org).

Normative data:

Structural connectome: https://datadryad.org/stash/dataset/doi:10.5061/dryad.nzs7h44q2

Functional connectome:
https://dataverse.harvard.edu/dataset.xhtml?persistentId=doi:10.7910/DVN/25833

Neurosynth database: https://github.com/neurosynth/neurosynth-data

Code availability

All code used to analyze the dataset is openly available within Lead-DBS/-Connectome software (https://github.com/leaddbs/leaddbs). Code to reproduce main results and figures is openly available on OSF (https://osf.io/bckuf).

Supplementary References

1. Wang, F. *et al.* In vivo human whole-brain Connectom diffusion MRI dataset at 760 μm isotropic resolution. *Sci. Data* **8**, 122 (2021).
2. Bt, Y. *et al.* The organization of the human cerebral cortex estimated by intrinsic functional connectivity. *J. Neurophysiol.* **106**, (2011).
3. Holmes, A. J. *et al.* Brain Genomics Superstruct Project initial data release with structural, functional, and behavioral measures. *Sci. Data* **2**, 150031 (2015).
4. Amunts, K., Mohlberg, H., Bludau, S. & Zilles, K. Julich-Brain: A 3D probabilistic atlas of the human brain's cytoarchitecture. *Science* **369**, 988–992 (2020).
5. Amaral, R. S. C. *et al.* Manual segmentation of the fornix, fimbria, and alveus on high-resolution 3T MRI: Application via fully-automated mapping of the human memory circuit white and grey matter in healthy and pathological aging. *Neuroimage* **170**, 132–150 (2018).
6. Edlow, B. L. *et al.* 7 Tesla MRI of the ex vivo human brain at 100 micron resolution. *Sci. Data* **6**, 244 (2019).
7. Neudorfer, C. *et al.* A high-resolution in vivo magnetic resonance imaging atlas of the human hypothalamic region. *Sci. Data* **7**, 305 (2020).
