# Supplementary material for: Optimal deep brain stimulation sites and networks for stimulation of the fornix in Alzheimer’s disease
Source: Nat Commun. 2022 Dec 14;13:7707. doi: 10.1038/s41467-022-34510-3 (PMC9751139; doi:10.1038/s41467-022-34510-3)
Supplement: Supplementary file 3 — Description of Additional Supplementary Files [file 41467_2022_34510_MOESM3_ESM.pdf]

Description of Additional Supplementary Files

Supplementary Movie 1. Demonstration of WarpDrive tool. Example of manual normalization refinement in the WarpDrive tool, a graphical interface that allows precise alignment of source and target landmarks by visualizing normalized image, with a template and atlas in MNI space, showing manual corrections of an example patient with focus on the fornix, using the atlas of the human hypothalamic region (Neudorfer et al. 2020). Followed by the visualization of the patient's electrode trajectory reconstruction after the pre-localization TRAC/CORE algorithm (Husch et al. 2018) and manual refinement (Oxenford et al. 2021). All tools available within Lead-DBS Toolbox (Horn et al. 2015).
Software available at https://github.com/netstim/SlicerNetstim
