# Supplementary material for: Optimal deep brain stimulation sites and networks for stimulation of the fornix in Alzheimer’s disease
Source: Nat Commun. 2022 Dec 14;13:7707. doi: 10.1038/s41467-022-34510-3 (PMC9751139; doi:10.1038/s41467-022-34510-3)
Supplement: Supplementary file 5 — Reporting Summary [file 41467_2022_34510_MOESM5_ESM.pdf]

nature research

Corresponding author(s): Andreas Horn

Last updated by author(s): Oct 5, 2022

Reporting Summary

Nature Research wishes to improve the reproducibility of the work that we publish. This form provides structure for consistency and transparency in reporting. For further information on Nature Research policies, see our Editorial Policies and the Editorial Policy Checklist.

Statistics

For all statistical analyses, confirm that the following items are present in the figure legend, table legend, main text, or Methods section.

n/a	Confirmed	
☐	☒	The exact sample size (n) for each experimental group/condition, given as a discrete number and unit of measurement
☐	☒	A statement on whether measurements were taken from distinct samples or whether the same sample was measured repeatedly
☐	☒	The statistical test(s) used AND whether they are one- or two-sided<br>*Only common tests should be described solely by name; describe more complex techniques in the Methods section.*
☒	☐	A description of all covariates tested
☒	☐	A description of any assumptions or corrections, such as tests of normality and adjustment for multiple comparisons
☐	☒	A full description of the statistical parameters including central tendency (e.g. means) or other basic estimates (e.g. regression coefficient) AND variation (e.g. standard deviation) or associated estimates of uncertainty (e.g. confidence intervals)
☐	☒	For null hypothesis testing, the test statistic (e.g. F, t, r) with confidence intervals, effect sizes, degrees of freedom and P value noted<br>*Give P values as exact values whenever suitable.*
☒	☐	For Bayesian analysis, information on the choice of priors and Markov chain Monte Carlo settings
☒	☐	For hierarchical and complex designs, identification of the appropriate level for tests and full reporting of outcomes
☐	☒	Estimates of effect sizes (e.g. Cohen's d, Pearson's r), indicating how they were calculated

Our web collection on statistics for biologists contains articles on many of the points above.

Software and code

Policy information about availability of computer code

Data collection	Microsoft Excel Version 16.65
Data analysis	MATLAB R2021a<br>Lead-DBS v2 (including tools: Lead-DBS Fiberfiltering, DBS Network mapping and Sweetspot Mapping, and adapted algorithms from SPM12, Advanced Normalization Tools, PaCER, Simbio, Fieldtrip): https://github.com/netstim/leaddbs, https://osf.io/bckuf/, https://github.com/netstim/SlicerNetstim<br>3D Slicer Version 5.0.3<br>Mango Version 4.1<br>Neurosynth decoder: https://github.com/neurosynth/neurosynth

For manuscripts utilizing custom algorithms or software that are central to the research but not yet described in published literature, software must be made available to editors and reviewers. We strongly encourage code deposition in a community repository (e.g. GitHub). See the Nature Research guidelines for submitting code & software for further information.

Data

Policy information about availability of data

All manuscripts must include a data availability statement. This statement should provide the following information, where applicable:

- Accession codes, unique identifiers, or web links for publicly available datasets
- A list of figures that have associated raw data
- A description of any restrictions on data availability

Anonymized derivatives of stimulation data used for the described analyses are openly available on OSF (https://osf.io/bckuf). The resulting tract atlas, sweet spot and fMRI network pattern are openly available within Lead-DBS software (www.lead-dbs.org).

Normative data:
Structural connectome: https://datadryad.org/stash/dataset/doi:10.5061/dryad.nzs7h44q2, Functional connectome: http://neuroinformatics.harvard.edu/gsp/
Neurosynth database: https://github.com/neurosynth/neurosynth-data

Field-specific reporting

Please select the one below that is the best fit for your research. If you are not sure, read the appropriate sections before making your selection.

- [x] Life sciences
- [ ] Behavioural & social sciences
- [ ] Ecological, evolutionary & environmental sciences

For a reference copy of the document with all sections, see nature.com/documents/nr-reporting-summary-flat.pdf

Life sciences study design

All studies must disclose on these points even when the disclosure is negative.

Sample size	46 subjects. The sample size was determined according to data availability from a phase I (NCT00658125) and a phase II (NCT01608061) clinical trials to evaluate the safety of fornix-Deep Brain Stimulation. A post-hoc power calculation for achieved power was performed with an effect size f^2 of 0.15 (moderate effect), power (1 - Beta error prob) of 0.73.
Data exclusions	2 subjects from NCT01608061 were excluded due to unavailability of imaging data.
Replication	Cross-Prediction across 2 subcohorts, Leave-one-out and K-fold cross validation
Randomization	Training and hold-out cohorts were determined in a randomized way.
Blinding	We estimated relationships between electrode placements and clinical improvements, rather than evaluating whether the surgical intervention is effective or not. The authors were, however, blinded to clinical data during imaging pre-processing and manual refinements to normalization and electrode localization.

Reporting for specific materials, systems and methods

We require information from authors about some types of materials, experimental systems and methods used in many studies. Here, indicate whether each material, system or method listed is relevant to your study. If you are not sure if a list item applies to your research, read the appropriate section before selecting a response.

Materials & experimental systems

n/a	Involved in the study
x	Antibodies
x	Eukaryotic cell lines
x	Palaeontology and archaeology
x	Animals and other organisms
	x Human research participants
	x Clinical data
x	Dual use research of concern

Methods

n/a	Involved in the study
x	ChIP-seq
x	Flow cytometry
	x MRI-based neuroimaging

Human research participants

Policy information about studies involving human research participants

Population characteristics	46 patients with mild Alzheimer's Disease (23 females, 67 ± 7.9 years old), diagnosed by expert clinicians with ADAS-cog 11 scores 12-24 points, and CDR of 0.5 or 1 intervened with Deep Brain Stimulation to the fornix were included.
Recruitment	Patients were retrospectively analyzed based on previously published data from clinical trials NCT00658125 and NCT01608061.
Ethics oversight	The study was approved by the ethics board of Charité – Universitaetsmedizin Berlin (master vote EA2/186/18). All procedures were carried out according to the declaration of Helsinki from 1975, participants signed an informed consent in person with the participation of a surrogate consenter.

Note that full information on the approval of the study protocol must also be provided in the manuscript.

Clinical data

Policy information about clinical studies

All manuscripts should comply with the ICMJE guidelines for publication of clinical research and a completed CONSORT checklist must be included with all submissions.

Clinical trial registration	Retrospective analysis of NCT00658125, NCT01608061.
Study protocol	N/A. Purely retrospective analysis of published data.
Data collection	N/A. Purely retrospective analysis of published data.
Outcomes	N/A. Purely retrospective analysis of published data.

Magnetic resonance imaging

Experimental design

Design type	Retrospective-Post hoc analysis
Design specifications	N/A
Behavioral performance measures	N/A

Acquisition

Imaging type(s)	Structural
Field strength	1.5 Tesla
Sequence & imaging parameters	Pre-op T1, T2-weighted MRI, Post-op T1-weighted MRI
Area of acquisition	Whole brain

Diffusion MRI ☐ Used ☒ Not used

Preprocessing

Preprocessing software	SPM12, ANTs, Lead-DBS, MATLAB
Normalization	ANTs
Normalization template	ICBM 2009b NLIN Asymmetric non-linear 2009b MNI152
Noise and artifact removal	Biasfield correction
Volume censoring	N/A

Statistical modeling & inference

Model type and settings	Mass univariate analysis, training -> hold-out cohort prediction, leave-one-out and k-fold cross-validations
Effect(s) tested	Cognitive improvement following DBS to the Fornix (as measured by changes on the ADAS-cog 11 scale).

Specify type of analysis: ☒ Whole brain ☐ ROI-based ☐ Both

Statistic type for inference (See Eklund et al. 2016)	training -> hold-out cohort prediction, leave-one-out and k-fold crossvalidations, predictive accuracy measured by Pearson- / Spearman cross-validations
Correction	FDR for flashback data analysis

Models & analysis

n/a	Involved in the study
☐	☒ Functional and/or effective connectivity
☒	☐ Graph analysis
☒	☐ Multivariate modeling or predictive analysis

Functional and/or effective connectivity	Spearman correlation, Pearson correlation
